# Mitochondrial Dysfunction and Neurodegenerative Disorders: Role of Nutritional Supplementation

**DOI:** 10.3390/ijms232012603

**Published:** 2022-10-20

**Authors:** David Mantle, Iain Parry Hargreaves

**Affiliations:** 1 Pharma Nord (UK) Ltd., Morpeth NE61 2DB, UK; 2 School of Pharmacy and Biomolecular Sciences, Liverpool John Moores University, Merseyside L3 5UX, UK

**Keywords:** mitochondrial dysfunction, neurodegenerative disorders, coenzyme Q10, B-vitamins, selenium, vitamin D, alpha-lipoic acid, l-carnitine

## Abstract

Mitochondrial dysfunction has been implicated in the pathogenesis of a number of neurodegenerative disorders, including Parkinson’s disease, Alzheimer’s disease, amyotrophic lateral sclerosis, multisystem atrophy, and progressive supranuclear palsy. This article is concerned specifically with mitochondrial dysfunction as defined by reduced capacity for ATP production, the role of depleted levels of key nutritionally related metabolites, and the potential benefit of supplementation with specific nutrients of relevance to normal mitochondrial function in the above neurodegenerative disorders. The article provides a rationale for a combination of CoQ10, B-vitamins/NADH, L-carnitine, vitamin D, and alpha-lipoic acid for the treatment of the above neurodegenerative disorders.

## 1. Introduction

Mitochondrial dysfunction is a common factor known to be involved in the pathogenesis of a number of both common (Parkinson’s disease, Alzheimer’s disease, amyotrophic lateral sclerosis) and less common (multisystem atrophy, progressive supranuclear palsy) neurodegenerative disorders, as detailed in subsequent sections of this article. Mitochondrial dysfunction in neurodegenerative disease is a broad area of research in which many review articles have been published. This article is concerned specifically with mitochondrial dysfunction as defined by reduced capacity for ATP production, with the objective of correlating evidence for the depletion of levels of key nutritionally-related mitochondrial metabolites, with the potential symptomatic benefit of their supplementation in the above neurodegenerative disorders; this in turn provides a rationale for the utilisation of multisupplement combinations for the management of these disorders, which to date has usually not been the case. Parkinson’s disease (PD) is a chronic and progressive neurodegenerative disorder characterised by proteinaceous intraneuronal Lewy body formation and striatal dopamine depletion in the substantia nigra of the mid brain [1]. Patients with PD commonly experience motor symptoms such as bradykinesia, tremor, muscle stiffness (rigidity), and postural instability. Alzheimer’s disease (AD) is a progressive neurodegenerative disorder characterised by initial memory impairment and cognitive decline. AD is the most common form of dementia and is characterised by an accumulation of abnormal neuritic plaques composed of β-amyloid peptide, together with neurofibrillary tangles of misfolded tau protein, in the brain of AD patients [2]. Amyotrophic lateral sclerosis (ALS), otherwise known as motor neuron disease, is a progressive disorder characterised by the degeneration of upper and lower motor neurons within the brain and spinal cord, resulting in a loss of muscle control. A hallmark of ALS is the development of ubiquinated protein aggregates [3]. Multiple system atrophy (MSA) is an example of one of the less common neurological disorders. MSA results from the progressive degeneration of neurons and glia, with the subsequent dysfunction of the autonomic nervous system. The pathogenesis of MSA has been linked to the dysfunction of an enzyme (COQ2; 4-parahydroxybenzoate: polyphenyl- transferase) in the CoQ10 synthetic pathway [4]. Progressive supranuclear palsy (PSP) is a disorder resulting from tau protein aggregation in brain tissue, causing problems with balance, movement, and vision [5]. In order to identify the most appropriate nutrients, one must first consider the mechanism of normal mitochondrial function, particularly with regard to cellular energy supply, and this is addressed in the following section. This review is structured to then include sections on the assessment of mitochondrial dysfunction, followed by sections on nutrient deficiency and nutrient supplementation (in the following order: CoQ10, selenium, B-vitamins/NADH, L-carnitine/acetyl-L-carnitine, alpha-lipoic acid, vitamin D3), respectively, in the above neurodegenerative disorders.

## 2. Normal Mitochondrial Function

Mitochondria have a number of key functions in cell metabolism, the most important of which is arguably cellular energy supply. Briefly, in the latter process, glucose (derived from dietary sugars) is converted via glycolysis into pyruvate, which is then imported into mitochondria. Within the mitochondrial matrix, the pyruvate is then converted via acetylCoA and the TCA cycle to carbon dioxide, generating NADH from NAD+ and FADH2 from FAD in the process [6]. As an alternative energy source, fatty acids (derived from dietary fats) can also be transported into mitochondria and thence into the TCA cycle. NADH (together with FADH2) then acts as the main source of electrons for transport along the electron transport chain in the process of oxidative phosphorylation, through which the majority of the cellular ATP requirement is generated [7]. It could be argued that a considerable number of nutrient-type substances are involved directly or indirectly with this aspect of mitochondrial function; however, the nutrients identified of principal importance in the present article include the B vitamins B1, B2, and B3 (a precursor of NADH), coenzyme Q10 (CoQ10), selenium and vitamin D, alpha lipoic acid, and L-carnitine. The B vitamins and alpha lipoic acid have important roles in the TCA cycle; CoQ10, selenium and vitamin D in oxidative phosphorylation; and L-carnitine in the transport of fatty acids into mitochondria prior to utilisation in the TCA cycle. Nutrients may have more than one function; for example, CoQ10 and alpha lipoic acid also act as important intracellular antioxidants.

## 3. Mitochondrial Dysfunction

As noted above, mitochondria have a number of important functions within cells, and a definition of mitochondrial dysfunction therefore depends on which of these functions is being addressed. In this article, mitochondrial dysfunction is defined as the inability to generate sufficient levels of ATP in response to cellular requirements. Based on this definition, mitochondrial dysfunction can be measured using a variety of systems and methods. Systems include isolated mitochondria, isolated cells, blood biomarkers, and real-time in vivo measurements [8,9]; measurement methods include quantification of electron transport complex levels or TCA cycle enzyme activities, assays to determine ATP levels, or assessment of ATP (or related metabolites) made via ^13^C NMR, ^31^P NMR, or positron emission spectroscopy [10,11]. Mitochondrial dysfunction may occur for a number of reasons, but this article is concerned specifically with mitochondrial dysfunction that results from a deficit in key nutritional substances, either dietary-derived or endogenously synthesised.

## 4. Evidence for Mitochondrial Dysfunction in Neurodegenerative Disorders

The common neurodegenerative disorders PD, AD, and ALS on which this article is focused are as recognised by Lamptey et al. [12].

In PD, dysfunction of the mitochondria resulting from alterations in mitochondrial morphology, mutations of mitochondrial DNA, anomalies in calcium homeostasis, and impairment of the electron transport chain have all been reported [13]. Mitochondrial dysfunction has been observed in both sporadic and genetic forms of PD, as well as toxin-induced models of the disease. In AD, mitochondrial dysfunction associated with altered mitochondrial morphology, decreased complex IV activity, and reduced ATP levels have been identified in postmortem tissue, in platelets, and in fibroblasts [14]. In ALS, disruption of mitochondrial structure, dynamics, bioenergetics, and calcium buffering have been extensively reported [3]. Analysis of mRNA expression levels using blood samples from ALS patients identified abnormalities in mitochondrial electron transport chain proteins, including reduced levels of FAD synthase, riboflavin kinase, cytochrome C1, and succinate dehydrogenase complex subunit B [15]. In multisystem atrophy (MSA), impaired respiratory chain complex activity/CoQ10 levels have been reported in postmortem brain tissue and in fibroblasts [16]. In progressive supranuclear palsy (PSP), mitochondrial DNA mutation and impairment of the electron transport chain have been reported [17].

## 5. Nutrient Deficiency in Neurodegenerative Disorders

Deficiencies of nutrient-type mitochondrial metabolites have been identified in PD, AD, ALS, MSA, and PSP, as described in the following sections; whether such deficiencies constitute a cause or consequence of these disorders remains to be established. These deficiencies are outlined in Table 1.

**Coenzyme Q10:** In PD, a deficiency in cerebral cortex CoQ10 status (together with impaired complex I activity) has been reported in PD patients [18,19]. A decrease in CoQ10 status has also been reported in both the plasma and platelets of PD patients [20,21]. Depleted levels of CoQ10 in blood are associated with an increased risk of developing AD [22]. In MSA, several studies have reported a reduction in plasma or postmortem brain tissue. Thus, in a series of 44 MSA patients, Mitsui et al. [23] found a significant reduction in the mean plasma CoQ10 level of approximately 30% compared to controls. Barca et al. [24] found CoQ10 levels to be significantly depleted (by 40%) in postmortem cerebellar tissue from MSA patients compared to controls. In addition, in a study using induced pluripotent stem cell (iPSC)-derived neurons, CoQ10 levels were significantly reduced in MSA patients, particularly those with COQ2 functional variants [25].

**Selenium:** Deficiency of selenium in brain tissue, via its role in selenoproteins such as the antioxidant enzyme glutathione peroxidase, has been implicated in the pathogenesis of PD [26]. Depletion of circulatory or brain tissue selenium levels are also implicated in the pathogenesis of AD [27,28]. In ALS, blood levels of selenium are inversely associated with ALS occurrence [29,30]. Selenium is an interesting case, as both reduced and excessive tissue levels have been implicated in the development of these neurodegenerative disorders [28,54].

**B-vitamins/NADH:** Reduced levels of vitamin B1 (thiamine) in blood and CSF have been reported in PD patients [31,32], and thiamine deficiency has been implicated in the pathogenesis of PD [33]. Vitamin B1 deficiency has been reported in blood and autopsied brain samples from AD patients [34]. In ALS patients, vitamin B1 levels are depleted in blood and CSF [35,36]. In a study comprising a large cohort of Chinese patients with ALS, Wang et al. [37] found that reduced blood levels of vitamin B2 (riboflavin) were associated with an increased risk of developing ALS. Reduced blood levels of vitamin B2 in PD patients were reported by Coimbra et al. [38], and similarly in AD patients by Liu et al. [39] and Lanyau-Dominguez et al. [40]. Levels of vitamin B3 (niacin), the precursor of NADH, are reduced in PD [41]. In AD, dietary intake of niacin was inversely related to the risk of cognitive decline and development of AD [42].

**l-carnitine/acetyl-l-carnitine:** l-carnitine blood levels were found to be significantly reduced in PD patients from the Faroe Islands, which have a high prevalence of PD [43]. Reduced levels of acyl-l-carnitine in plasma from PD patients have been reported [44,45]. Lodeiro et al. [46] reported reduced levels of l-carnitine in CSF from early AD cases. Reduced levels of carnitine acetyltransferase (the catalyst of l-carnitine acylation to acetyl-L-carnitine) have been reported in postmortem brain tissue from AD patients [47].

**Vitamin D3:** Vitamin D receptors (VDR) are located within mitochondria and are necessary for normal mitochondrial function [55]. Studies in animal model systems have shown VDR knockdown results in decreased mitochondrial oxidative capacity and reduced ATP production [56]. There is evidence for depleted circulatory vitamin D3 levels and increased risk of PD [48], AD [49,50], and ALS [51]. In the less common neurodegenerative disorders, vitamin D3 deficiency has been reported in MSA [52,53].

## 6. Nutrient Supplementation in Neurodegenerative Disorders

**Coenzyme Q10:** In a Phase II clinical trial conducted by Schults et al. [57], oral CoQ10 supplementation (300–1200 mg/day) was found to reduce the functional decline of patients with early-stage PD. A subsequent Phase III clinical trial involving six hundred patients was undertaken with PD patients receiving CoQ10 dosages of 1200 or 2400 mg/d [58]. Despite 1200 mg/d being the highest dosage used in the previous study, the mean change in UPDRS (Unified Parkinson’s Disease Rating Scale) score of treated patients was not found to be significantly lower than that of the placebo group; the researchers concluded that since CoQ10 appeared to show no apparent clinical benefit, they could not recommend its use in the treatment of early-stage PD. The contrasting findings of the clinical studies by Shults et al. and Beal et al. may reflect the broad range of sporadic PD patients used in the two clinical trials, with the heterogeneous patient populations contributing to their contradictory findings. Furthermore, no assessment of an underlying CoQ10 deficiency was determined in the PD patients prior to commencing CoQ10 supplementation in the study by Beal et al., which may explain the limited therapeutic potential of CoQ10 reported.

In AD, in a randomised clinical trial in which 70 patients with mild-to-moderate AD were treated with CoQ10 (400 mg; three times/day) for 16 weeks, no clinical benefit or significant effect on the CSF biomarkers for AD (amyloid-beta and tau protein levels) were reported [59]. To date, no large clinical studies have assessed the cognitive effect of CoQ10 supplementation in AD; however, clinical studies with the CoQ10 analogue idebenone have reported modest cognitive and behavioural improvements in patients following supplementation [60,61]. There were, however, drop-out rates as high as 71% in these studies. A subsequent, one-year, multicentre, double blind, placebo-controlled, randomised clinical trial of 536 AD patients found that idebenone treatment (120, 240, or 360 mg, three times a day) failed to slow cognitive decline in AD [62].

In ALS, although supplemental CoQ10 or its synthetic analogue MitoQ prolonged survival in a mouse model of ALS [63,64], a Phase II trial supplementing 2700mg CoQ10/day for 9 months in 185 ALS patients found insufficient benefit to warrant a Phase III study [65].

To date, there have been no randomised controlled trials of CoQ10 in MSA.

There have been two randomised controlled trials of supplementary CoQ10 in PSP. In the study by Stamelou et al. [66] of 20 PSP patients, supplementation with 5mg/kg/day CoQ10 for 6 weeks resulted in improved cerebral energy metabolism assessed via magnetic resonance spectroscopy, as well as an improvement in PSP rating scale. In the study by Apetauerova et al. [67] of 60 PSP patients, supplementation with 2400mg/day CoQ10 for up to 12 months did not significantly improve PSP symptoms or disease progression; however, the study had a high patient dropout rate and lacked the precision to exclude a moderate benefit of CoQ10.

**Selenium:** Selenium supplementation partially reversed impaired dopaminergic neurotransmission in MPTP-induced PD in mice [68] but has not been evaluated clinically in randomised controlled trials. In contrast, there have been several randomised controlled trials supplementing selenium (typically 200mcg/day) in AD. In the PREADVISE trial, selenium alone or in combination with vitamin E had no significant effect in reducing AD incidence [69], whereas supplementation with selenium in combination with probiotics showed some improvement in cognitive function [70]. Cardoso et al. [71] reported that supplementation with high-dose sodium selenate significantly improved Mini-Mental State score in AD subjects.

**NADH/NAD/nicotinamide:** There have been two randomised controlled trials of NADH/NAD/nicotinamide—in PD and AD, respectively. In a Phase I study, 30 PD patients were given 1gm of nicotinamide riboside or placebo for 30 days; this resulted in increased brain levels of NAD and upregulated mitochondrial metabolism (as measured via 31P nmr spectroscopy and positron emission tomography), which was associated with mild clinical improvement [72]. In AD, patients were administered 10mg NADH/day or placebo for 6 months; subjects treated with NADH showed no evidence of progressive cognitive deterioration and had significantly higher total scores on the MDRS (Mattis Dementia Rating Scale) compared with subjects treated with placebo (*p* < 0.05). Analysis of MDRS subscales revealed significantly better performance by NADH subjects on measures of verbal fluency (*p* = 0.019) and visual–constructional ability (*p* = 0.038) [73].

**B-vitamins:** A clinical study comprising 50 PD patients found that long-term treatment with vitamin B1 (100mg administered via intramuscular injection) improved PD symptoms, particularly motor function [74]. There have been several clinical studies supplementing vitamin B1, or its synthetic derivatives benfotiamine or fursultiamine, in AD. Supplementation with vitamin B1 (3 g/day for 3 months to 1 year) in AD patients showed mixed results; the study by Nolan et al. [75] reported no benefit on AD progression, whist the study by Meador et al. [76] found some symptomatic benefit. A randomised controlled trial supplementing benfotiamine (600mg/day for 1 year) reported improved cognitive function in AD patients [77], and a study supplementing fursultiamine (100mg/day for 3 months) resulted in improved cognitive function in cases with mild AD [78]. With regard to vitamin B2, oral supplementation (90mg/day for 6 months) resulted in improved motor capacity in a series of 20 PD patients [38]. In a case study, a patient with a form of ALS resulting from riboflavin transporter deficiency showed dramatic symptomatic improvement following high-dose oral supplementation (15mg/kg) with riboflavin [79].

**l-carnitine:** Beneficial effects of l-carnitine or acetyl-l-carnitine have been described in several animal models of Parkinson’s disease—in MPTP-induced Parkinson’s disease in mice, acetyl-l-carnitine protected against damage to endothelial cells and loss of dopaminergic neurons in the substantia nigra pars compacta and caudate putamen [80]. Acetyl-l-carnitine also protected the dopaminergic nigrostriatal pathway in a 6-hydroxydopamine-induced model of Parkinson’s disease in the rat [81]. To date, there have been no randomised controlled trials of l-carnitine or acetyl-l-carnitine in PD patients.

There have been nine randomised controlled trials of L-carnitine or acetyl-l-carnitine (typically 1.5–3 gm/day for 6–12 months) in AD. Administration of acetyl-l-carnitine to AD patients by Spagnoli et al. [82] resulted in a slower rate of deterioration in 13 of the 14 outcome measures, reaching statistical significance for the Blessed Dementia Scale, logical intelligence, verbal critical abilities, long-term verbal memory, and selective attention. Sano et al. [83] found that administration of acetyl-l carnitine significantly slowed the decline in memory-related parameters. Pettegrew et al. [84] assessed the effect of acetyl-l-carnitine administration via ^31^P NMR finding an increase in ATP levels and significantly less deterioration in AD patient Mini-Mental Status and Alzheimer’s Disease Assessment Scale test scores. Thal et al. [85] and Brooks et al. [86] reported that acetyl-l-carnitine slowed the progression of AD (assessed via the Alzheimer Disease Assessment Scale) in younger subjects. A meta-analysis by Montgomery et al. [87] confirmed the efficacy of acetyl-l-carnitine for the treatment of mild cognitive impairment and mild Alzheimer’s disease. l-carnitine or acetyl-l-carnitine have been shown to improve manifestations of AD in animal models [88,89]. Kepka et al. [90] have reviewed the potential role of dietary l-carnitine in the prevention of Alzheimer’s disease.

There has been one randomised controlled trial of acetyl-l-carnitine in ALS [91]. Forty-two patients received acetyl-l-carnitine and forty received the placebo, with the following parameters assessed: number of patients no longer self-sufficient; changes in ALSFRS-R, MRC, FVC, and McGill Quality of Life (QoL) scores; median survival. In the cohort receiving acetyl-L-carnitine, 34 patients became non-self-sufficient versus 30 receiving placebo (*p* = 0.0296). Mean ALSFRS-R scores at 48 weeks were 33.6 and 27.6 (*p* = 0.0388), respectively, and mean FVC scores 90.3 and 58.6 (*p* = 0.0158), respectively. Median survival was 45 months (acetyl-l-carnitine) and 22 months (placebo) (*p* = 0.0176).

***Alpha*-lipoic acid:** The neuroprotective action of *alpha* lipoic acid has been demonstrated in a number of cellular or animal models of PD [92,93,94]. To date, there have been no randomised controlled trials or other types of clinical studies of *alpha*-lipoic acid in PD.

With regard to AD, in a randomised controlled trial, Shinto et al. [95] found that alpha-lipoic acid and omega-3 fatty acids in combination slowed cognitive and functional decline in AD patients over 12 months. In an open-label clinical study, 600 mg of alpha lipoic acid was given daily to 43 patients with AD (receiving a standard treatment with acetylcholinesterase inhibitors) over a period of 48 months. Patients were assessed via Mini-Mental State examination, AD assessment scale, and cognitive subscale. Whilst the improvement in patients with moderate dementia was not significant, the disease progressed extremely slowly in patients with mild dementia [96]. An open-label study by Fava et al. [97] evaluated the effect of alpha lipoic acid (600 mg/day) on cognitive function in AD patients, with and without diabetes. One hundred and twenty-six patients with AD were divided into two groups, with (group A) or without (group B) diabetes. Cognitive functions were assessed by MMSE, Alzheimer’s Disease Assessment Scale–Cognitive (ADAS-Cog), Clinician’s Interview-Based Impression of Severity (CIBIC), Clinical Dementia Rating (CDR), and Alzheimer’s Disease Functional and Change Scale (ADFACS). At the end of the study, MMSE scores showed a significant improvement in 43% of patients in group A (26 subjects) and 23% in group B (15 subjects) compared to baseline (*p* = 0.001). ADAS-Cog, CIBIC, and ADFACS scores also showed a significant improvement in group A versus group B. Studies performed in animal models of memory loss associated with aging and AD have shown that α-LA improves memory in a variety of behavioural paradigms [98].

A randomised controlled clinical trial to assess the neuroprotective effect of alpha-lipoic acid in ALS is currently in progress, led by Dr Zhiying Wu of Zhejiang University Medical School, China.

**Vitamin D3:** There have been several randomised controlled trials of vitamin D3 supplementation in PD. Suzuki et al. [99] reported that vitamin D3 supplementation might stabilise PD for short periods, whilst a meta-analysis by Zhou et al. [100] found no evidence for improved motor function in PD patients. Supplementation with vitamin D3 reduced the risk of osteopenia in PD [101]. A randomised controlled trial by Jia et al. [102] suggested that vitamin D3 supplementation may improve cognitive decline in AD patients, whilst a meta-analysis by Du et al. [103] found no evidence that supplementation with vitamin D3 reduced the risk of developing AD. Vitamin D3 supplementation had no significant effect on motor dysfunction in ALS patients [104].

Details of the use of nutrient supplementation in neurodegenerative diseases are summarised in Table 2.

## 7. Conclusions

In reviewing the potential benefits of nutritional supplements on mitochondrial dysfunction in neurodegenerative disorders, one must first define mitochondrial dysfunction and how it is measured, and secondly identify the key nutrients of relevance to mitochondrial function. For the purposes of this review, mitochondrial dysfunction is defined in terms of altered cellular energy production, with the key nutrients identified as CoQ10, selenium, B vitamins/NADH, l-carnitine, alpha-lipoic acid, and vitamin D. The potential role of these nutrients was then reviewed in selected neurodegenerative disorders, which are characterised by mitochondrial dysfunction; PD, AD and ALS as examples of common disorders; and MSA and PSP as examples of less common disorders. Whilst there is considerable evidence for the efficacy of all of the above nutrients in cell-based or animal models of these disorders, relatively few relevant clinical studies have been identified. The best-studied nutrient/disease combinations were for CoQ10 and PD (six randomised controlled trials), and acetyl-l-carnitine in AD (nine randomised controlled trials); there have also been single randomised controlled trials of CoQ10 in ALS and acetyl-l-carnitine in ALS, respectively. To date, there have only been two randomised controlled trials of NADH/nicotinamide (in PD and AD, respectively), and one randomised control of alpha-lipoic acid in AD. In some of these studies, the outcome has been surprisingly disappointing, a notable example being the lack of efficacy of CoQ10 in a Phase III trial in PD. This in turn may be a reflection of the current uncertainty as to whether such substances can access the blood–brain barrier; however, this may also relate to the fact that nutrients are usually used in isolation in clinical studies, and that combinations of the above nutrients may be more effective. An example is the synergistic interaction between CoQ10 and selenium. CoQ10 occurs in cells in two closely related forms: oxidised (ubiquinone) and reduced (ubiquinol); continual interconversion between these CoQ10 forms is required for normal mitochondrial function, including cellular energy generation and antioxidant protection. Selenium is a component of the enzyme thioredoxin reductase, which catalyses the reduction of ubiquinone to ubiquinol. Thus, a deficiency of either selenium or CoQ10 can impact on this interconversion process, and subsequent mitochondrial function or supplementation with CoQ10 is likely to be less effective if individuals are also deficient in selenium [104]. Examples of this type of approach in clinical situations include the combination of CoQ10 and selenium for the prevention of cardiovascular disease in the elderly [105], CoQ10 and NADH used to improve fatigue in chronic fatigue syndrome [106], CoQ10 and L-carnitine for migraine prophylaxis [107], and L-carnitine and alpha-lipoic acid for the improvement of peripheral neuropathy in diabetic patients [108]. The study by Cornelli [109] is of particular interest, since some improvement in cognitive function (assessed via MMSE score) in AD patients was noted after supplementation with a combination of B vitamins, vitamins C and E, CoQ10, carnosine, beta-carotene, selenium, and l-cysteine. In this review, we have selected nutrients known to have a key role in mitochondrial function; however, a limitation to this approach is the exclusion of other nutrients known to be involved in mitochondrial metabolism. 

In summary, we have selected a number of neurodegenerative disorders that are known to involve mitochondrial dysfunction in their pathogenesis. We have further selected a number of nutrients that have a key role in mitochondrial function. We then correlated data on the deficiency (Table 1) and supplementation of these nutrients (Table 2) in the said neurodegenerative disorders. In this review, we have therefore provided a rationale for a combination of CoQ10, B-vitamins/NADH, l-carnitine, vitamin D, and alpha-lipoic acid to support the future treatment of these neurodegenerative disorders. To the best of our knowledge, this is the first review to systematically correlate evidence for the depletion and potential symptomatic benefit of these key mitochondrial metabolites in this range of neurodegenerative disorders.

## Figures and Tables

**Table 1:** Nutritional deficiencies in neurodegenerative disorders.

Nutrient	Report of Deficiency	Ref No.
Coenzyme Q10	Deficiency in cerebral cortex CoQ10 status in PD patients Reduced CoQ10 in plasma and platelets in PD patients Depleted levels of CoQ10 in blood associated with development of AD Reduced CoQ10 in plasma or postmortem brain tissue of MSA patients	[18,19] [20,21] [22] [23,24,25]
Selenium	Se deficiency in brain tissue in PD patients Depleted Se levels in blood or brain tissue in AD Se blood levels inversely associated with ALS	[26] [27,28] [29,30]
B-vitamins/NADH	Reduced B1 levels in blood and CSF fluid in PD patients B1 deficiency in blood and autopsied brain samples from AD patients B1 levels depleted in blood and CSF in ALS Reduced blood B2 levels associated with developing ALS Reduced blood B2 in PD patients Reduced blood B2 in AD patients Reduced B3 (niacin) in PD Niacin intake inversely related to development of AD	[31,32,33] [34] [35,36] [37] [38] [39,40] [41] [42]
l-carnitine/acetyl-l-carnitine	Reduced blood levels in PD Reduced CSF levels in AD Reduced levels of carnitine acetyltransferase in postmortem brain tissue of AD patients	[43,44,45] [46] [47]
PVitamin D_3_	Depleted D3 blood levels and increased risk of PD Depleted D3 blood levels and increased risk of AD Depleted D3 blood levels and increased risk of ALS D3 deficiency reported in MSA	[48] [49,50] [51] [52,53]

**Table 2:** Nutritional supplementation in neurodegenerative disorders.

Supplement	Effect	Ref No.
Coenzyme Q10	300–1200 mg/day reduced the functional decline of patients with early-stage PD 1200 or 2400 mg/day did not lower the mean change in UPDRS 400 mg three times/day showed no clinical benefit or significant effect on the CSF biomarkers for AD 2700 mg/day found insufficient benefit to warrant a Phase III study in ALS patients 5 mg/kg/day resulted in improved cerebral energy metabolism and in improvement in PSP rating scale (PSP = progressive supranuclear palsy) 2400 mg/day did not significantly improve PSP symptoms or disease progression	[57] [58] [59] [65] [66] [67]
Selenium	No RCTs in PD patients Se alone or in combination with vitamin E had no significant effect in reducing AD incidence Se in combination with probiotics showed some improvement in cognitive function in AD subjects Se significantly improved Mini Mental State score in AD subjects	[68] [69] [70] [71]
NADH/NAD/Nicotinamide	1 g/day nicotinamide riboside resulted in increased brain levels of NAD and upregulated mitochondrial metabolism in PD patients 10mg NADH/day was associated with no evidence of progressive cognitive deterioration and with significantly higher total scores on the Mattis Dementia Rating Scale as well as significantly better scores in verbal fluency and visual-constructional ability	[72] [73]
B-vitamins	Long-term treatment with vitamin B_1_ (intramuscular injection 100 mg) improved PD symptoms, particularly motor function Mixed results of vitamin B_1_ treatment in AD patients: no effect on AD progression vs. some symptomatic benefit Benfotiamine (600 mg/day for 1 year) improved cognitive function in AD patients Fursultiamine (100 mg/day for 3 months) improved cognitive function in cases with mild AD Vitamin B2 (90mg/day for 6 months) resulted in improved motor capacity in 20 PD patients High-dose oral (15mg/kg) of riboflavin in a patient with a form of ALS resulting from riboflavin transporter deficiency showed dramatic symptomatic improvement following a case study	[74] [75,76] [77] [78] [38] [79]
l-carnitine/acetyl-l-carnitine	To date, no RCTs of l-carnitine or acetyl-l-carnitine in PD patients, only preclinical studies Slower rate of deterioration in 13 of the 14 outcome measures on the Blessed Dementia Scale in AD patients Significant slowing of the decline in memory-related parameters in AD patients Increase in ATP levels and less deterioration in Mini-Mental Status and AD Assessment Scale test scores Slower progression of AD assessed via the Alzheimer Disease Assessment Scale in younger subjects Meta-analysis confirmed the efficacy of acetyl-l-carnitine for the treatment of mild cognitive impairment and mild AD One RCT of acetyl-l-carnitine in ALS: mean ALSFRS-R scores at 48 weeks were 33.6 and 27.6, respectively; mean FVC scores 90.3 and 58.6, respectively; median survival was 45 months (acetyl-l-carnitine) and 22 months	[80,81] [82] [83] [84] [85,86] [87] [91]
*alpha*-lipoic acid	Neuroprotective action of *alpha*-lipoic acid demonstrated in cellular and animal models of PD; to date, no RCTs or other clinical studies of *alpha*-lipoic acid in PD. Combined *alpha*-lipoic acid and omega-3 fatty acids slowed cognitive and functional decline in AD patients 600 mg/day slowed the progression of AD over 48 months 600 mg/day improved cognitive function in AD	[92,93,94] [95] [96] [97]
Vitamin D_3_	D_3_ supplementation might stabilise PD for short periods Meta-analysis found no evidence for improved motor function in PD D_3_ supplementation reduced the risk of osteopenia in PD D3 supplementation may improve cognitive decline in AD patients Meta-analysis found no evidence that D_3_ supplementation reduced the risk of developing AD D_3_ supplementation had no significant effect on motor dysfunction in ALS patients	[99] [48] [100] [101] [102] [103]

## Data Availability

Not applicable.
